# Anti-Inflammatory and Antioxidant Effects of Topical Formulations Containing Plant Extracts, Methylsulfonylmethane, and Peptiskin^®^ in In Vitro Models of Arthritis

**DOI:** 10.3390/ph18091270

**Published:** 2025-08-26

**Authors:** Thi Xoan Hoang, Nhat Minh Dang, Kang Gyu Bae, Jae Young Kim

**Affiliations:** 1 Department of Life Science, Gachon University, Seongnam 13120, Republic of Korea; xoanht@ntt.edu.vn (T.X.H.); dangnhatminh.ump@gmail.com (N.M.D.); 2 NTT Hi-Tech Institute, Nguyen Tat Thanh University, Ho Chi Minh City 700000, Vietnam; 3 Cosmetic Devision, 3H Co., Ltd., Daegu 41059, Republic of Korea; kgbae@3hk.co.kr

**Keywords:** AS632, anti-inflammatory, antioxidant, arthritis, macrophages, keratinocytes, chondrocytes, ROS, cytokines, MMP

## Abstract

**Background**: This study aimed to evaluate the anti-inflammatory and antioxidant effects of AS632 and AS633, two topical formulations composed of natural plant-derived ingredients, for potential use in arthritis therapy. **Methods**: AS632 and AS633 were formulated with natural plant extracts—including *Punica granatum* seed oil, *Gaultheria procumbens* essential oil, *Centella asiatica* extract, and *Camellia sinensis* extract—and methylsulfonylmethane (MSM). AS632 additionally contained a peptide-based component, Peptiskin^®^. Both formulations were tested in THP-1-derived macrophages, HaCaT keratinocytes, and C28/I2 chondrocytes stimulated with lipopolysaccharide (LPS) or pro-inflammatory cytokines. **Results**: Both formulations significantly reduced TNF-α, IL-1β, matrix metalloproteinase (MMP-2, MMP-9, MMP-13) expression, and ROS level, with AS632 showing greater suppression of TNF-α in macrophages compared to AS633. In addition, both formulations demonstrated cytoprotective effects against cytokine-induced damage in chondrocytes. **Conclusions**: AS632 and AS633 are promising topical candidates for managing arthritis and chronic inflammatory skin or joint disorders.

## 1. Introduction

Arthritis is a prevalent inflammatory disorder characterized by joint pain, swelling, and cartilage degradation, ultimately leading to impaired mobility and a decreased quality of life [1]. The pathogenesis of arthritis involves complex interactions among immune cells, inflammatory cytokines, oxidative stress, and matrix metalloproteinases (MMPs), all of which contribute to disease progression [2].

Current treatments, such as nonsteroidal anti-inflammatory drugs (NSAIDs) and corticosteroids, offer symptomatic relief but are associated with significant side effects and limited efficacy in halting disease progression [3]. Due to the anatomical, immunological, and pathophysiological complexity of arthritis, effective management requires more than just the suppression of inflammation. Treatment strategies must also address structural restoration, including cartilage protection, inhibition of bone erosion, and control of synovial hyperplasia [4].

Merely eliminating inflammation is insufficient, as arthritis inevitably progresses to structural damage and functional impairment over time [5]. Synovial hyperplasia (pannus formation) and cartilage/bone erosion are hallmark pathological features that cannot be effectively controlled by conventional anti-inflammatory agents such as NSAIDs [4]. While acute inflammatory conditions, like dermatitis, may respond to short-term immunosuppression, chronic immune modulation is essential in the treatment of arthritis [5].

Management of arthritis often necessitates the long-term use of disease-modifying agents such as methotrexate and tumor necrosis factor (TNF) inhibitors, which require careful monitoring of infection risks, drug resistance, and systemic side effects associated with immunosuppressive therapies [6]. Therefore, there is an urgent need for novel therapeutic agents that can not only suppress inflammation and oxidative stress but also promote joint homeostasis.

Natural and synthetic small molecules with anti-inflammatory and antioxidant properties have been extensively investigated as potential treatments for arthritis. Plant-derived compounds such as *Punica granatum* seed oil [7,8], *Gaultheria procumbens* leaf essential oil (wintergreen oil, steam-distilled) [9], *Centella asiatica* extract [10], and *Camellia sinensis* extract [11] have shown potent anti-inflammatory and antioxidative effects. These findings suggest their therapeutic potential in managing chronic inflammatory diseases such as arthritis. Dimethyl sulfone, commonly known as methylsulfonylmethane (MSM), is often combined with plant extracts in arthritis treatments due to its complementary mechanisms, which enhance therapeutic efficacy [12]. Peptiskin^®^, a bioactive ingredient developed by the Solabia Group, contains oligopeptides derived from L-lysine and L-arginine. These peptides are known to stimulate collagen synthesis while inhibiting collagen degradation by downregulating MMP activity [13].

In this study, we formulated a new formula, AS632, by combining plant extracts, MSM, and Peptiskin^®^ with soy lecithin and glyceryl stearate as emulsifying agents to enhance both stability and efficacy. To compare the role of Peptiskin^®^, we also prepared a control formulation, AS633, which is identical to AS632 in all components except for the absence of Peptiskin^®^. We evaluated the anti-inflammatory and antioxidant properties of AS632 and AS633 in multiple cell types relevant to arthritis, including macrophages, keratinocytes, and chondrocytes. Specifically, we investigated their effects on pro-inflammatory cytokine production, reactive oxygen species (ROS) accumulation, and matrix metalloproteinase (MMP) activity. Our findings provide compelling evidence that both AS632 and AS633 may represent viable therapeutic candidates for the treatment of arthritis.

## 2. Results

### 2.1. Particle Sizes and Zeta Potentials of AS632 and AS633

The particle sizes and zeta potentials of AS632 and AS633 were measured to confirm their nanoscale formulation and colloidal stability. As shown in Table 1, AS632 exhibited an average particle size of 239.0 nm, whereas AS633 showed a slightly smaller size of 226.7 nm. Both values fall within the optimal range (<400 nm) for effective transdermal delivery. The zeta potential values were −46.3 mV for AS632 and −50.0 mV for AS633, indicating excellent dispersion stability, as colloidal particles with zeta potentials exceeding ±30 mV are generally considered stable.

### 2.2. Cytotoxicity Analysis of AS632 and AS633

To determine safe and effective working concentrations for AS632 and AS633, we evaluated the effects of a range of concentrations (100 µL/mL (1:10 dilution), 10 µL/mL (1:100 dilution), 1 µL/mL (1:1000 dilution), and 0.1 µL/mL (1:10,000 dilution)) on cell viability in three cell types: THP-1 macrophages, human chondrocytes, and keratinocytes.

Our results showed that in THP-1-derived macrophages, higher concentrations (100 µL/mL and 10 µL/mL) of AS632 and AS633 significantly reduced cell viability, indicating cytotoxic effects, whereas 1 µL/mL and 0.1 µL/mL concentrations were well tolerated. In contrast, in chondrocytes C228/I2 and keratinocytes HaCaT, treatment with AS632 100 µL/mL and 10 µL/mL concentrations promoted cell proliferations. AS633 did not exhibit notable cytotoxicity in any of the tested cell types, but its effect on cell proliferation was also less pronounced compared to AS632.

**Figure 1:**
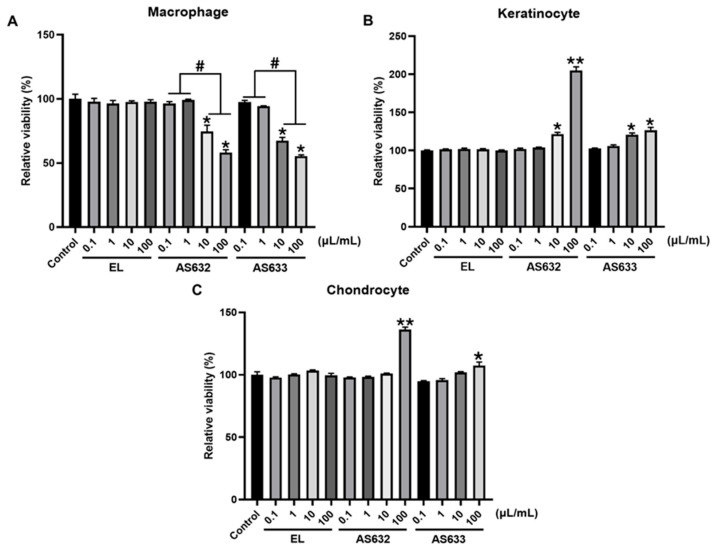
Effect of AS632 and AS633 on cell viability. THP-1-derived macrophages (**A**), keratinocytes HaCaT (**B**), and chondrocytes (**C**) C28/I2 were treated with different concentrations of empty liposomes (ELs), AS632, or AS633 for 24 h. Cell viability was assessed by MTT assay (EZ-Cytox, DOGEN). * *p* < 0.05, ** *p* < 0.01 vs. control; # *p* < 0.05.

Based on these findings, 1 µL/mL concentration was selected for subsequent experiments, as it represented a consistent balance between safety and bioactivity across different cell types. These results also suggest a potential role for AS632 in enhancing wound healing and tissue regeneration, particularly in keratinocytes and chondrocytes (Figure 1).

### 2.3. Anti-Inflammatory and Antioxidant Effects of AS632 in Macrophages

**Figure 2:**
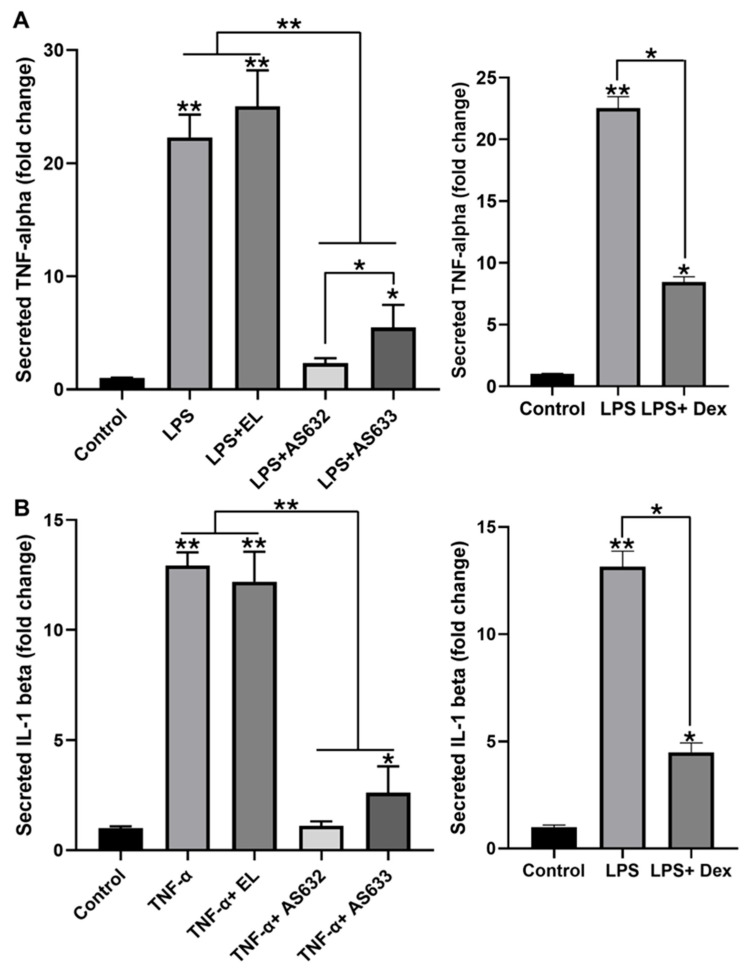
Inhibitory effect of AS632 on pro-inflammatory cytokine secretion in LPS-stimulated macrophages. THP-1 monocytes were differentiated into M0 macrophages by treatment with 100 ng/mL PMA for 48 h. Cells were then stimulated with 100 ng/mL LPS for 24 h, followed by treatment with either empty liposomes (ELs), AS632, or AS633 at a concentration of 1 µL/mL or 1 μM dexamethasone as a positive control for an additional 48 h. Culture supernatants were collected, and the levels of TNF-α (**A**) and IL-1β (**B**) were quantified using ELISA. * *p* < 0.05; ** *p* < 0.01 compared to the LPS-treated control group.

To evaluate the anti-inflammatory potential of AS632 and AS633, we assessed their ability to suppress pro-inflammatory cytokine production in LPS-stimulated THP-1-derived macrophages. ELISA analysis revealed that LPS stimulation significantly increased TNF-α and IL-1β secretion by more than 20-fold and 10-fold, respectively, compared to the untreated control. Treatment with AS632 or AS633 significantly reduced the levels of both cytokines, whereas control liposomes had no inhibitory effect (Figure 2). Notably, AS632 was more effective than AS633 in suppressing TNF-α secretion (*p* < 0.05), suggesting an enhanced anti-inflammatory effect potentially mediated by its unique component, Peptiskin^®^.

**Figure 3:**
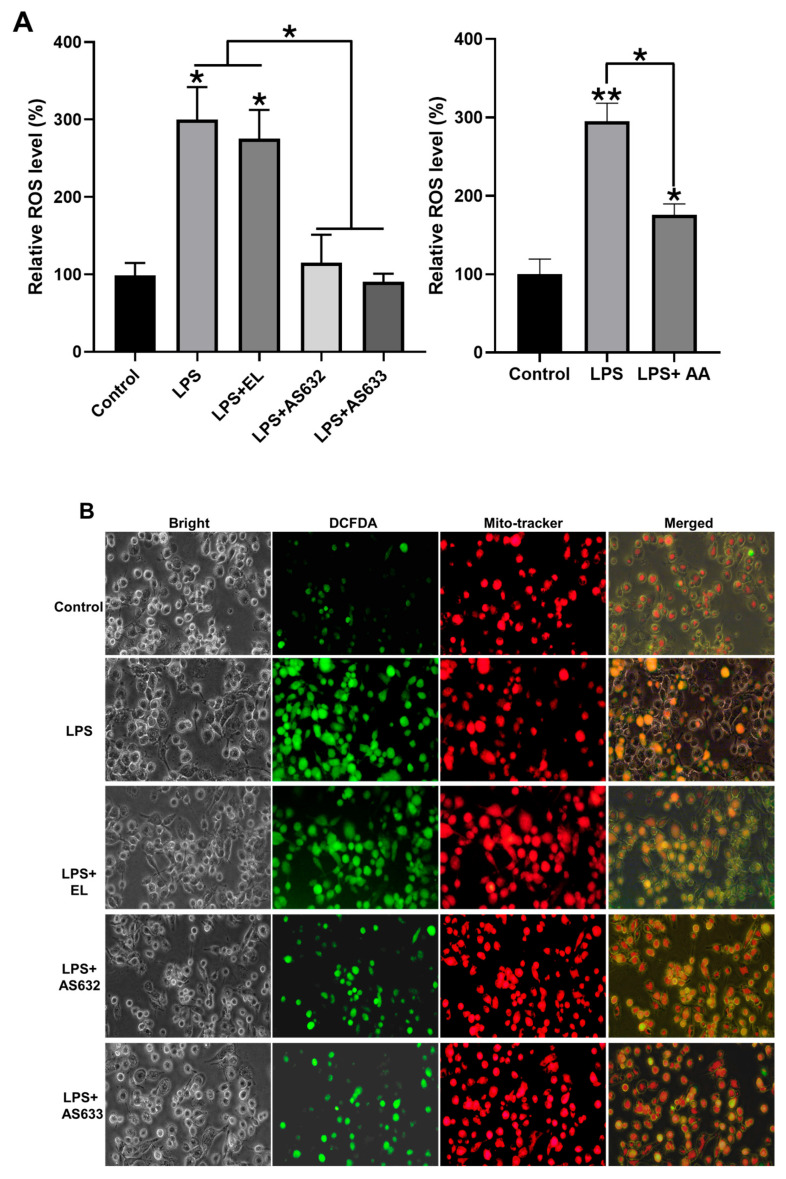
Inhibitory effect of AS632 on LPS-induced ROS production in macrophages. THP-1 monocytes were differentiated into M0 macrophages by treatment with 100 ng/mL PMA for 48 h. Cells were then stimulated with 100 ng/mL LPS for 24 h, followed by treatment with empty liposomes (ELs), AS632, or AS633 at a concentration of 1 µL/mL or 2 µM ascorbic acid as a positive control for an additional 48 h. Intracellular ROS production was assessed by staining with DCFDA. ROS levels were quantified by flow cytometry (**A**) and visualized using fluorescence microscopy at 200× magnification (**B**). Green fluorescence indicates DCFDA-stained ROS, and red fluorescence represents MitoTracker-stained mitochondria. * *p* < 0.05; ** *p* < 0.01 compared to the LPS-treated control.

Reactive oxygen species (ROS) are key mediators in LPS-stimulated macrophages. We next examined whether AS632 and AS633 modulate ROS generation. Following LPS stimulation, cells were treated with AS632, AS633, or control liposomes for 48 h. Flow cytometry showed that LPS significantly increased intracellular ROS levels, which were markedly reduced by both AS632 and AS633 (Figure 3A). This result was further corroborated by fluorescence microscopy (Figure 3B). There was no statistically significant difference between the two formulations in ROS reduction.

**Figure 4:**
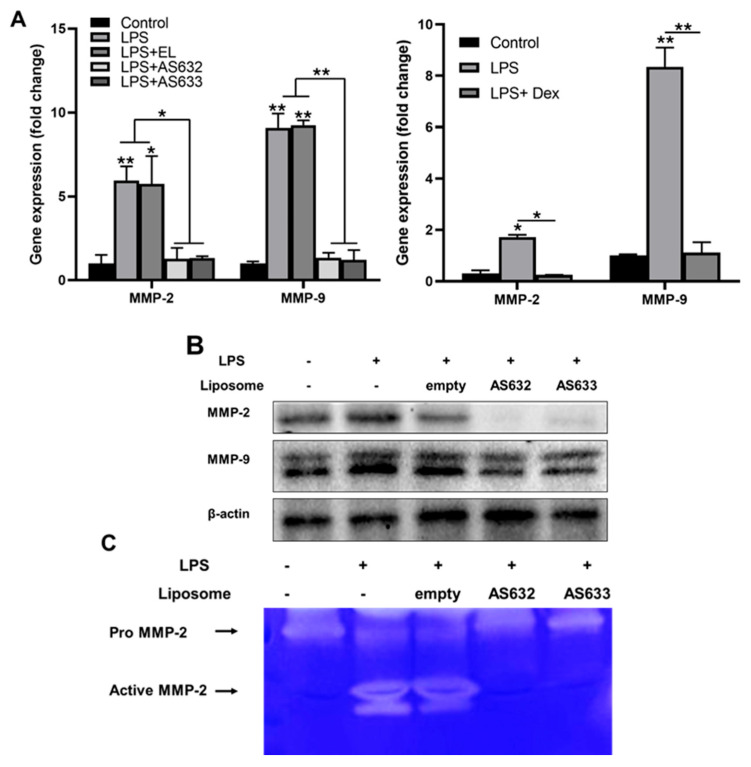
AS632 and AS633 suppress MMP-2 and MMP-9 expression and activity in LPS-stimulated macrophages. THP-1 monocytes were differentiated into M0 macrophages by treatment with 100 ng/mL PMA for 48 h. Cells were then stimulated with 100 ng/mL LPS for 24 h, followed by treatment with empty liposomes (ELs), AS632, or AS633 at a concentration of 1 µL/mL or 1 μM dexamethasone as a positive control. (**A**) Gene expression: After 6 h of treatment, total RNA was extracted, and the mRNA expression levels of MMP-2 and MMP-9 were analyzed by real-time qPCR. (**B**) Protein expression: After 48 h of treatment, cells were lysed, and the protein levels of MMP-2 and MMP-9 were evaluated by Western blotting. (**C**) Enzymatic activity: Culture supernatants were collected, concentrated, and analyzed by gelatin zymography to assess MMP gelatinase activity. * *p* < 0.05; ** *p* < 0.01 compared to the LPS-treated control group.

We also investigated the effects of AS632 and AS633 on the expression and activity of matrix metalloproteinases (MMPs). Real-time PCR demonstrated that the mRNA levels of MMP-2 and MMP-9 were significantly downregulated by AS632 and AS633 treatment (Figure 4A). Western blot analysis revealed a parallel reduction in protein expression (Figure 4B), and gelatin zymography confirmed the suppression of MMP-2 enzymatic activity (Figure 4C) by AS632 and AS633 compared to LPS-treated and control liposome groups.

### 2.4. Anti-Inflammatory and Antioxidant Effects of AS632 in Keratinocytes

**Figure 5:**
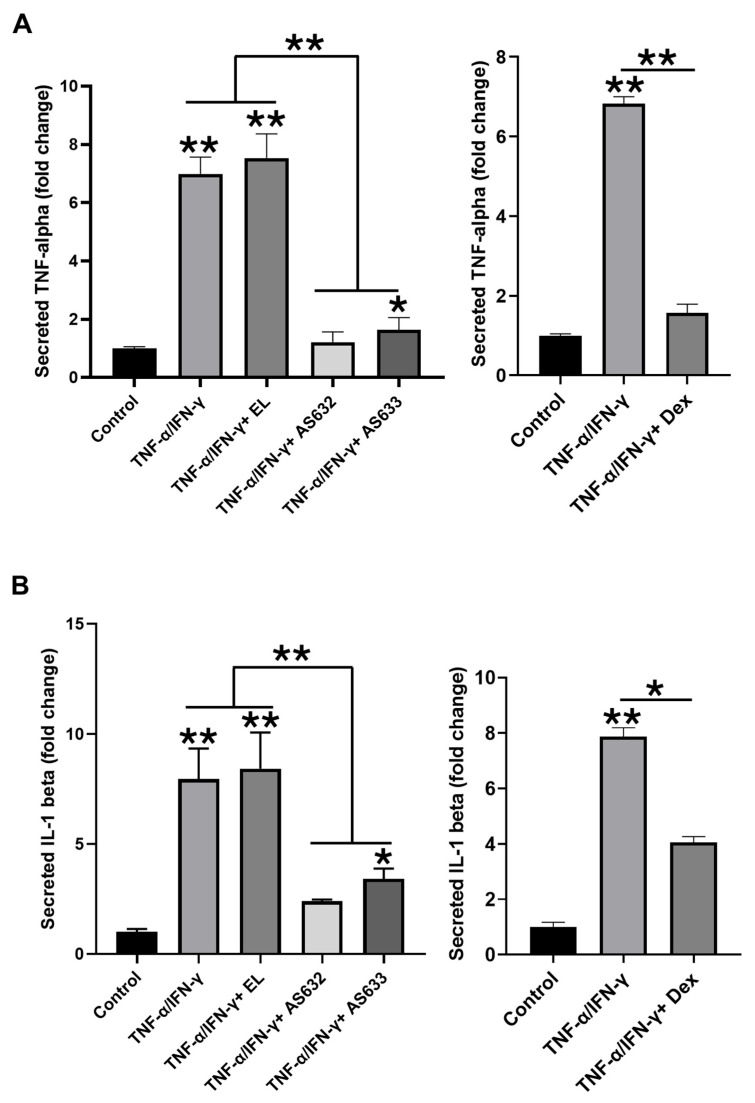
Inhibitory effect of AS632 on pro-inflammatory cytokine secretion in IFN-γ/TNF-α-stimulated HaCaT keratinocytes. HaCaT keratinocytes were stimulated with 10 ng/mL IFN-γ and 10 ng/mL TNF-α for 24 h, followed by treatment with empty liposomes (ELs), AS632, or AS633 at a concentration of 1 µL/mL or 1 μM dexamethasone as a positive control for 48 h. Culture supernatants were then collected, and the levels of pro-inflammatory cytokines TNF-α (**A**) and IL-1β (**B**) were measured using ELISA. * *p* < 0.05; ** *p* < 0.01 compared to the cytokine-stimulated control group.

To evaluate the effects of AS632 and AS633 on keratinocytes, HaCaT cells were stimulated with TNF-α and IFN-γ, followed by treatment with either AS632, AS633, or control liposomes. ELISA results showed that cytokine stimulation significantly increased the secretion of TNF-α and IL-1β, which was significantly inhibited by both AS632 and AS633 (Figure 5).

**Figure 6:**
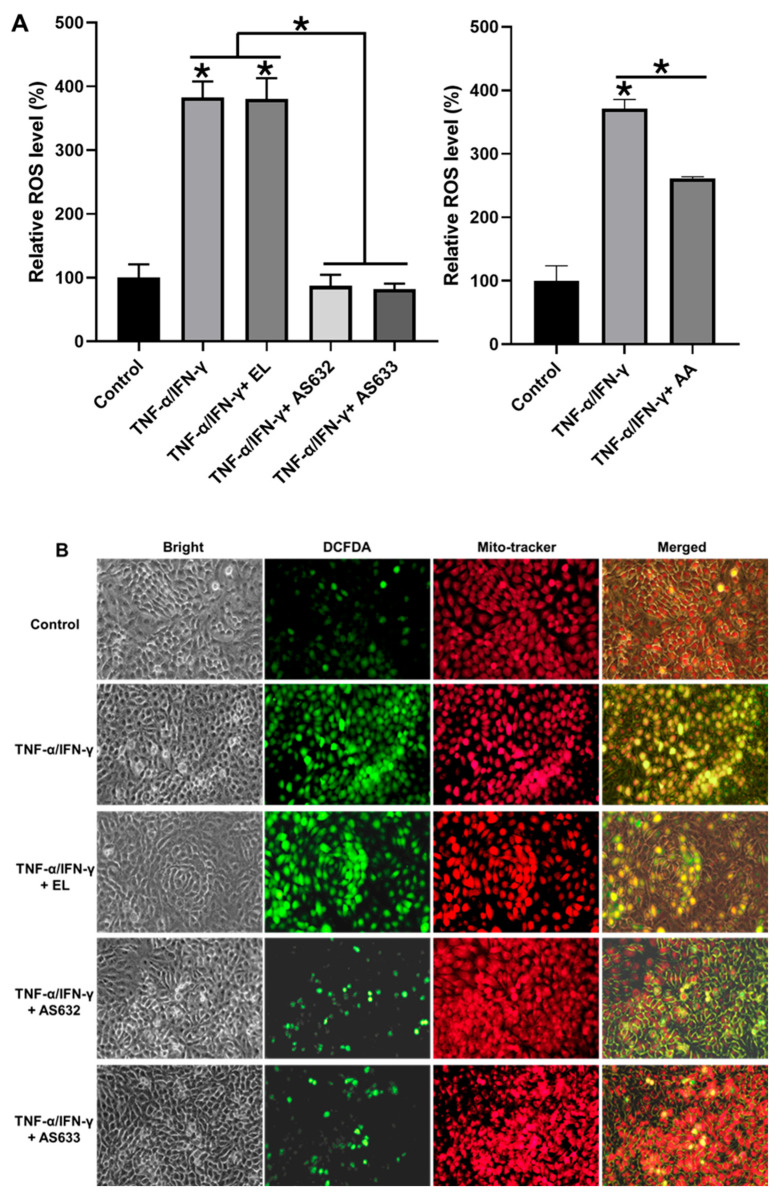
Antioxidant effect of AS632 on TNF-α/IFN-γ-induced ROS production in HaCaT keratinocytes. HaCaT cells were stimulated with 10 ng/mL IFN-γ and 10 ng/mL TNF-α for 24 h, followed by treatment with empty liposomes (ELs), AS632, or AS633 at a concentration of 1 µL/mL or 2 µM ascorbic acid as a positive control for 48 h. Intracellular ROS levels were assessed by DCFDA staining. ROS production was quantified by flow cytometry (**A**) and visualized by fluorescence microscopy at 200× magnification (**B**). Green fluorescence indicates DCFDA-stained ROS, and red fluorescence represents mitochondria stained with MitoTracker. * *p* < 0.01 compared to the cytokine-treated control.

We further examined ROS levels in stimulated HaCaT cells. As shown in Figure 6, TNF-α/IFN-γ treatment, with or without control liposomes, led to a substantial increase in ROS generation. In contrast, AS632 and AS633 treatment significantly reduced intracellular ROS levels, indicating their antioxidant capacity in keratinocytes.

### 2.5. Anti-Inflammatory and Antioxidant Effects of AS632 in Chondrocytes

**Figure 7:**
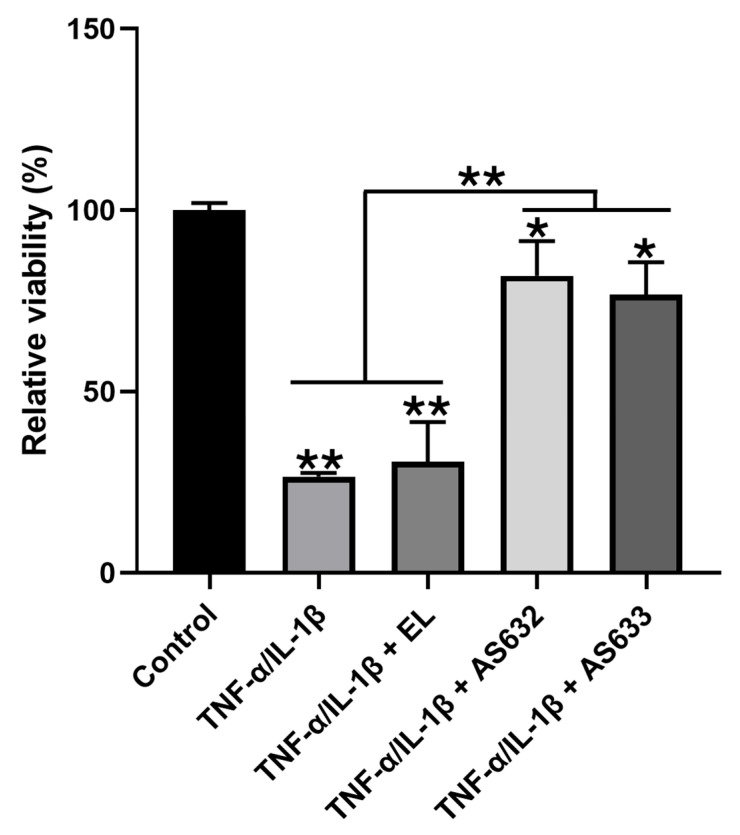
Protective effect of AS632 against TNF-α/IFN-γ-induced cytotoxicity in C28/I2 chondrocytes. C28/I2 chondrocytes were pre-treated with empty liposomes (ELs), AS632, or AS633 at a concentration of 1 µL/mL for 2 h, followed by stimulation with 1 ng/mL IL-1β and 10 ng/mL TNF-α for 48 h. Cell viability was assessed using the EZ-Cytox reagent according to the manufacturer’s instructions. * *p* < 0.05; ** *p* < 0.01 compared to the cytokine-treated control.

To assess the protective effects of AS632 and AS633 in chondrocytes, we pre-treated C28/I2 cells with either the compound or control liposomes for 2 h, followed by stimulation with IL-1β and TNF-α. Cell viability analysis showed that cytokine treatment significantly reduced cell viability, whereas AS632 and AS633 pre-treatment markedly preserved cell survival (Figure 7).

**Figure 8:**
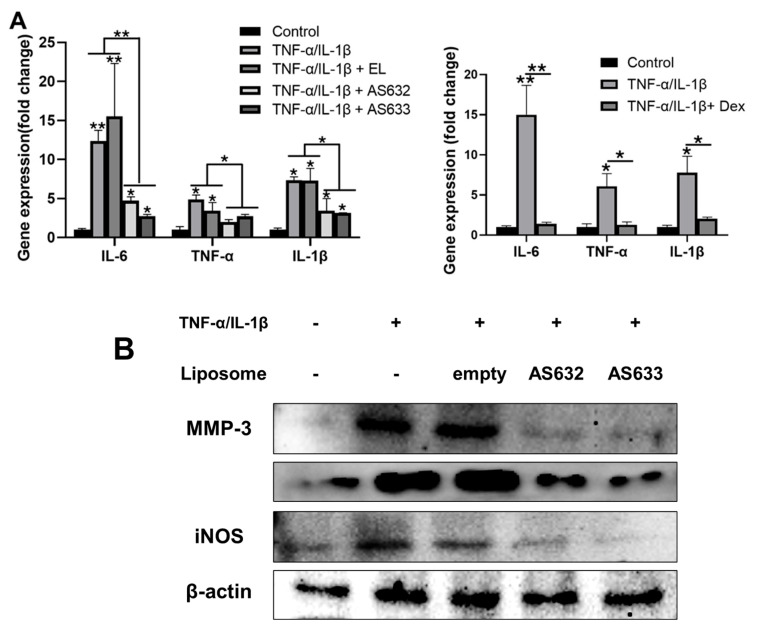
Protective effect of AS632 against TNF-α/IL-1β-induced inflammatory responses in C28/I2 chondrocytes. C28/I2 chondrocytes were pre-treated with empty liposomes (ELs), AS632, or AS633 at a concentration of 1 µL/mL or 1 μM dexamethasone as a positive control for 2 h, followed by stimulation with 1 ng/mL IL-1β and 10 ng/mL TNF-α. (**A**) After 6 h of treatment, total RNA was extracted, and the mRNA expression levels of IL-6, TNF-α, and IL-1β were analyzed by real-time qPCR. (**B**) After 24 h of treatment, cells were lysed, and protein levels of MMP-3, MMP-13, and iNOS were analyzed by Western blotting. * *p* < 0.05; ** *p* < 0.01 compared to the cytokine-treated control group.

We then investigated the effects of AS632 and AS633 on inflammatory gene and protein expression in chondrocytes. Compared with the untreated control, IL-1β/TNF-α treatment significantly upregulated mRNA levels of IL-6, TNF-α, and IL-1β. Pre-treatment with AS632 or AS633 significantly downregulated these genes (Figure 8A). Correspondingly, Western blot analysis revealed that the protein levels of inducible nitric oxide synthase (iNOS), MMP-3, and MMP-13—enzymes associated with cartilage matrix degradation [14]—were elevated following cytokine stimulation but were effectively suppressed by treatment with AS632 and AS633 (Figure 8B).

**Figure 9:**
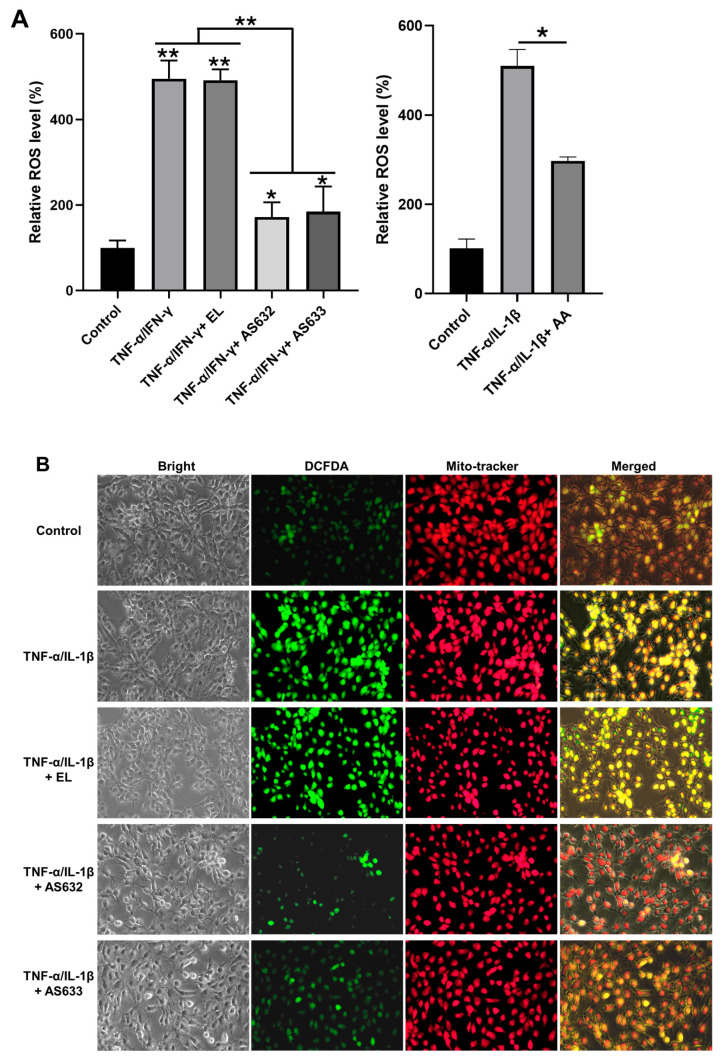
Effect of AS632 on TNF-α/IL-1β-induced ROS production in C28/I2 chondrocytes. C28/I2 chondrocytes were pre-treated with empty liposomes (ELs), AS632, or AS633 at a concentration of 1 µL/mL or 2 µM ascorbic acid as a positive control for 2 h, followed by stimulation with 1 ng/mL IL-1β and 10 ng/mL TNF-α for 24 h. Intracellular ROS production was assessed by DCFDA staining. ROS levels were quantified by flow cytometry (**A**) and visualized using fluorescence microscopy at 200× magnification (**B**). Green fluorescence indicates DCFDA-stained ROS, and red fluorescence indicates mitochondria stained with MitoTracker. * *p* < 0.05; ** *p* < 0.01 compared to the cytokine-treated control.

Finally, ROS production in C28/I2 chondrocytes was assessed using DCFDA staining. Cytokine stimulation resulted in more than a 4-fold increase in total cellular ROS compared to the untreated control. However, AS632 and AS633 significantly inhibited this ROS elevation (Figure 9), confirming their antioxidant properties in protecting chondrocytes from inflammation-induced oxidative stress.

## 3. Discussion

The natural ingredients incorporated into AS632 and AS633 were selected based on their complementary mechanisms targeting inflammation, oxidative stress, and extracellular matrix degradation, all of which are central to arthritis pathophysiology. *Punica granatum* (pomegranate) seed oil is rich in punicic acid and polyphenols, known to suppress pro-inflammatory cytokines and promote tissue regeneration [7,8]. *Gaultheria procumbens* (wintergreen) leaf essential oil contains methyl salicylate, a natural salicylate analog with established analgesic and anti-inflammatory effects frequently used in musculoskeletal disorders [9,15]. *Centella asiatica* whole plant extract was used for its triterpenoid saponins, including asiaticoside and madecassoside, which promote collagen synthesis and reduce inflammation in joint and dermal tissue [10,16,17]. *Camellia sinensis* (green tea) leaf extract is a source of catechins such as EGCG, well known for their strong antioxidant capacity and MMP inhibitory activity. The combination of these botanicals was designed to synergistically address both inflammatory and degenerative processes associated with arthritis [11].

The particle characterization results support the suitability of AS632 and AS633 for topical application. Both formulations exhibited nanoscale particle sizes, which are advantageous for enhanced skin penetration and cellular uptake [18,19]. In addition, their high zeta potential values (greater than −45 mV) indicate strong electrostatic repulsion between particles, minimizing aggregation and ensuring colloidal stability during storage and biological application [19]. These physicochemical properties likely contribute to the observed biological efficacy and underscore the potential of AS632 and AS633 as stable and effective anti-inflammatory agents.

This study demonstrates that AS632 possesses significant anti-inflammatory and antioxidant properties across multiple cell types relevant to arthritis pathogenesis. In macrophages, AS632 treatment effectively suppressed LPS-induced TNF-α and IL-1β secretion, indicating a strong inhibitory effect on pro-inflammatory signaling pathways [20]. Since activated macrophages are critical drivers of synovial inflammation in arthritis [21], these findings highlight the therapeutic promise of AS632 for inflammatory joint diseases.

Beyond its anti-inflammatory actions, AS632 showed robust antioxidant activity by reducing intracellular ROS levels in both macrophages and keratinocytes. Given that ROS play a central role in oxidative stress-mediated synovial damage and cartilage degradation [22], the ability of AS632 to attenuate ROS accumulation suggests a protective mechanism against oxidative joint damage.

To better understand the cytocompatibility and potential proliferative effects of AS632 and AS633, we evaluated their cytotoxicity effects across a concentration range (0.1–100 µL/mL). At higher concentrations (10 and 100 µL/mL), both AS632 and AS633 induced cytotoxicity in THP-1 derived macrophages. However, only AS632 promoted significant cell proliferation in chondrocytes and keratinocytes, whereas AS633 showed no notable effects on cell viability or proliferation across the tested concentrations. These findings suggest that AS632 may offer dual therapeutic benefits—mitigating inflammation while simultaneously supporting tissue regeneration, particularly in skin and joint tissue. Based on its consistent safety and efficacy across cell types, the concentration of 1 µL/mL was selected for subsequent experiments.

The enhanced biological effects observed with AS632, compared to AS633, are likely attributed to the inclusion of Peptiskin^®^ in AS632. Peptiskin^®^, composed of L-arginine/L-lysine oligopeptides, has been shown to inhibit MMP-1 activity and promote synthesis of decorin and collagens I/III/V [23,24,25,26,27,28]. These actions may account for the ability of AS632 to suppress TNF-α secretion in macrophages and to promote cell proliferation in keratinocytes and chondrocytes, as observed in our study.

Consistent with these effects, AS632 also significantly reduced the expression and enzymatic activity of MMP-2 and MMP-9 in macrophages and MMP-3 and MMP-13 in chondrocytes—enzymes critically involved in cartilage degradation in arthritis joints [29,30,31]. The inhibition of these MMPs suggests that Peptiskin^®^ not only supports extracellular matrix stabilization but may also act synergistically with plant-derived anti-inflammatory ingredients to reinforce chondroprotective outcomes.

Additionally, AS632 demonstrated cytoprotective effects in chondrocytes exposed to IL-1β/TNF-α stimulation, as evidenced by improved cell viability and reduced expression of inflammatory mediators such as iNOS and MMP-3. These effects are particularly relevant to arthritis pathogenesis, where oxidative stress and pro-inflammatory signaling contribute to cartilage degeneration [32,33].

Although AS632 and AS633 exhibited comparable performance in several assays—such as ROS suppression and general anti-inflammatory activity—AS632 consistently demonstrated superior efficacy in key functional parameters. This differential efficacy underscores the potential of Peptiskin^®^ as a bioactive component that amplifies AS632’s therapeutic effects by targeting multiple inflammatory and matrix-degeneration pathways. While the current study did not isolate the effect of Peptiskin^®^ independently, future investigations involving Peptiskin^®^-only formulations or peptide-depleted AS632 variants would help clarify its mechanistic contributions. Mechanistic studies exploring peptide-specific signaling responses, including NF-κB translocation and SMAD phosphorylation, are also warranted.

**Figure 10:**
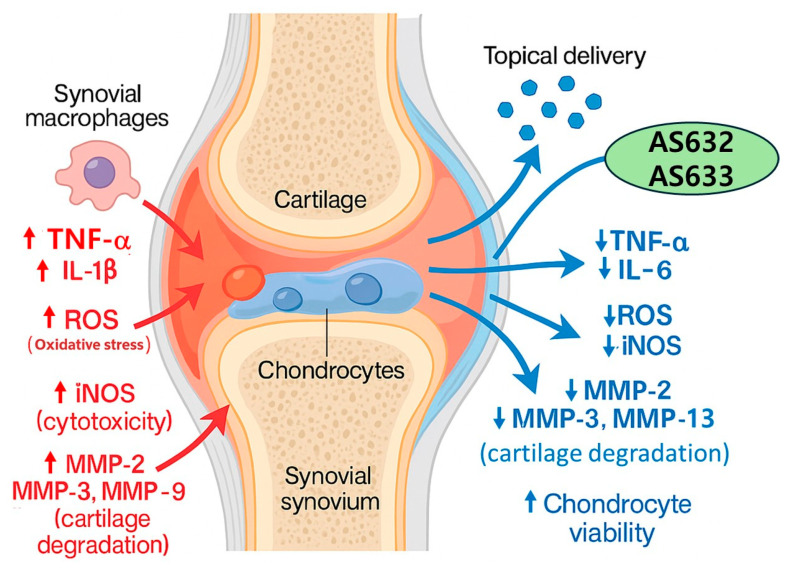
Schematic illustration of the joint-protective mechanisms of AS632 and AS633 in an arthritic joint. This diagram depicts the proposed mechanisms of AS632 and AS633 based on joint tissue structure. In the inflamed joint (**left**), macrophages and chondrocytes are activated by IL-1β and TNF-α, leading to increased production of pro-inflammatory cytokines, ROS, and matrix metalloproteinases (MMPs), ultimately resulting in cartilage degradation and cellular cytotoxicity. On the (**right**), treatment with AS632 reduces cytokine production, oxidative stress, and MMP expression while enhancing chondrocyte viability and preserving cartilage integrity. Effects of AS632 and AS633 on macrophages, keratinocytes, and chondrocytes suggest a multi-target mechanism beneficial for restoring joint homeostasis in arthritis.

Collectively, our results indicate that AS632 and AS633 may serve as a promising therapeutic agent for arthritis by simultaneously targeting inflammation, oxidative stress, and matrix degradation (Figure 10). Future investigations should focus on elucidating the precise molecular mechanisms underlying AS632’s effects and evaluating its therapeutic efficacy and safety in in vivo models of arthritis.

## 4. Materials and Methods

### 4.1. Chemicals and Antibodies

Phorbol 12-myristate 13-acetate (PMA; Cat. No. Ab120297) and the DCFDA/H2DCFDA Cellular ROS Assay Kit (Cat. No. Ab113851) were obtained from Abcam (Cambridge, UK). The EZ-Cytox Enhanced Cell Viability Assay Kit was purchased from DOGEN (Seoul, Republic of Korea). Lipopolysaccharide (LPS; Cat. No. L2630), dexamethasone (Dex. Cat. No. D4902), and ascorbic acid (AA. Cat. No. 255564) were purchased from Sigma-Aldrich (St. Louis, MO, USA). Antibodies against iNOS (Cat. No. sc-7271) and MMP-9 (Cat. No. sc-13520) were acquired from Santa Cruz Biotechnology (Dallas, TX, USA), while the MMP-3 antibody (Cat. No. LS-C27030-200) was obtained from LS Bio (Seattle, WA, USA). The Gelatin Zymography Kit (Cat. No. PMC-AK47) was sourced from Cosmo Bio (Tokyo, Japan). Recombinant human TNF-α protein (Cat. No. 10291-TA-01M) was purchased from R&D Systems (Minneapolis, MN, USA). The ELISA MAX^TM^ Deluxe Set for Human IL-1β (Cat. No. 437004) was obtained from BioLegend (San Diego, CA, USA). MitoTracker^TM^ Deep Red was purchased from Thermo Fisher Scientific (Waltham, MA, USA).

### 4.2. Plant Extract Preparation

All plant materials used in the formulation were sourced from GMP-certified industrial suppliers (Cosmax NBT, Seoul, Republic of Korea) using cultivated material. The plant origin and species authentication were verified by the supplier based on macroscopic and HPLC fingerprinting analysis. Although no voucher specimens were deposited in an academic herbarium due to industrial sourcing, botanical authentication records and quality certificates are maintained in the manufacturer’s in-house archives and can be made available upon request.

*Centella asiatica* extract was prepared from the dried whole plant using 70% aqueous ethanol at room temperature for 24 h, followed by filtration and rotary evaporation. The extract contained 40–50% total triterpenoid saponins (asiaticoside, madecassoside, madecassic acid), with a typical yield of 45 mg/g dry weight. *Camellia sinensis* leaf extract was produced by 50% ethanol extraction of dried green tea leaves. Catechin content was ≥200 mg/g as measured by UV–Vis at 540 nm, with an overall yield of ~60 mg/g. *Gaultheria procumbens* essential oil was obtained by steam distillation of fermented leaves (≥3 days), yielding >96% methyl salicylate. *Punica granatum* seed oil was cold-pressed without solvent, yielding 25–30% oil based on seed weight.

### 4.3. Preparation of AS632 and AS633

AS632 was formulated by dissolving 2.5% (*w*/*w*) of *Punica granatum* seed oil, 0.5% (*w*/*w*) of *Gaultheria procumbens* leaf essential oil (wintergreen oil, steam-distilled, 0.5% (*w*/*w*) of *Centella asiatica* extract, 0.5% (*w*/*w*) of dimethyl sulfone, 0.01% (*w*/*w*) of *Camellia sinensis* leaf extract, and 0.08% (*w*/*w*) of Peptiskin^®^. Emulsifiers included 1.125% (*w*/*w*) soy lecithin and 0.5% (*w*/*w*) glyceryl stearate, mixed into a solvent system consisting of 4.7% (*w*/*w*) butylene glycol, 3.0% (*w*/*w*) ethanol, 0.75% (*w*/*w*) glycerin, and 83.7749% (*w*/*w*) purified water. The mixture was homogenized using a microfluidizer, followed by the addition of 2.0% (*w*/*w*) 1,2-hexanediol as a preservative.

AS633 was prepared identically to AS632, except that 0.08% (*w*/*w*) of Peptiskin^®^ was omitted and replaced with 0.08% (*w*/*w*) of purified water.

Control empty liposomes (ELs) were prepared using identical emulsifiers (soy lecithin and glyceryl stearate) and the solvent system but without any plant extracts, MSM, or Peptiskin^®^.

The qualitative and quantitative composition of AS632 and AS633 was defined by precise weight/weight ratios during formulation, verified by gravimetric control. Active compounds from each extract or oil were confirmed using supplier-provided analytical certificates (HPLC for asiaticoside and catechins; GC for methyl salicylate; UV for total polyphenols). Due to proprietary formulation protocols, batch-specific quantitative validation was not performed beyond verification of active marker presence using chromatographic identity matching.

### 4.4. Particle Size and Zeta Potential Analysis

The particle size and zeta potential of AS632 and AS633 were measured using an ELSZ-2000 Zeta-Potential & Particle Size Analyzer (Otsuka Electronics, Osaka, Japan) based on laser light scattering. AS632 had an average particle size of 239 nm, and AS633 measured 226.7 nm. Both values fall within the optimal range (<400 nm) for efficient skin penetration. The zeta potentials were −46.3 mV for AS632 and −50.0 mV for AS633, indicating excellent colloidal stability (values greater than ±30 mV are considered stable).

### 4.5. Cell Culture and Treatment

The test formulations were applied at a final concentration of 1 μL/mL (1:1000 dilution) unless otherwise stated. Ascorbic acid (2 μM) was used as a positive control for antioxidant assays (ROS inhibition), and dexamethasone (1 μM) was included in cytokine inhibition assays for comparison.

The human monocytic cell line THP-1 (ATCC) was cultured in an RPMI-1640 medium supplemented with 10 mM HEPES, 10% heat-inactivated fetal bovine serum (FBS), 0.05 mM β-mercaptoethanol, and 1% antibiotic-antimycotic. Cells were maintained at 37 °C in a humidified 5% CO_2_ incubator. Differentiation into macrophages was induced by treating THP-1 cells with 100 ng/mL PMA for 48 h. The resulting macrophages were then exposed to 100 ng/mL LPS for 24 h, followed by treatment with AS632, AS633, control empty liposomes, or positive controls for 48 h.

HaCaT keratinocytes (ATCC) and C28/I2 chondrocytes (Sigma-Aldrich) were cultured in DMEM supplemented with 10% heat-inactivated FBS and 1% penicillin-streptomycin. HaCaT cells were stimulated with 10 ng/mL TNF-α and 10 ng/mL IFN-γ for 24 h, followed by treatment with AS632, AS633, control liposomes, or positive controls for 48 h.

C28/I2 cells were pre-treated with AS632, AS633, control liposomes, or positive controls for 2 h and then stimulated with 1 ng/mL IL-1β and 10 ng/mL TNF-α. Treatment duration was 48 h for cytotoxicity evaluation and 24 h for other assays.

### 4.6. Cytotoxicity Testing by MTT Assay

THP-1-derived macrophages, HaCaT keratinocytes, and C28/I2 chondrocytes were seeded into 96-well plates at 2 × 10^4^ cells/well and incubated overnight. Cells were treated with AS632 or AS633 at various dilutions (0.1, 1, 10, and 100 μL/mL). Cell viability was assessed using the MTT assay (EZ-Cytox, DOGEN, Seoul, Republic of Korea). After treatment, 10 µL of MTT reagent was added per well and incubated at 37 °C for 3 h. Absorbance was measured at 450 nm using a microplate reader. Viability was expressed as a percentage of the untreated control.

### 4.7. Enzyme-Linked Immunosorbent Assay (ELISA)

Supernatants were collected from treated cultures to quantify secreted IL-1β and TNF-α. For IL-1β, concentrations were measured using a commercial ELISA kit (BioLegend, San Diego, CA, USA) following the manufacturer’s protocol, and absorbance was read at 450 nm.

For TNF-α, supernatants were coated onto ELISA plates and incubated overnight at 4 °C. After blocking with 5% bovine serum albumin (BSA), plates were incubated with a primary anti-TNF-α antibody for 4 h followed by an HRP-conjugated secondary antibody. Absorbance was measured at 450 nm.

### 4.8. RNA Preparation and Real-Time Quantitative PCR

Total RNA was extracted using the easy-BLUE^TM^ Total RNA Extraction Kit (iNtRON Biotechnology, Seongnam-si, Republic of Korea). Concentration was determined using a MaestroNano Spectrophotometer (Maestrogen, Las Vegas, NV, USA).

For cDNA synthesis, 2 µg of RNA was reverse-transcribed using Hyperscript^TM^ 2X RT Master Mix (Geneall Biotechnology, Seoul, Republic of Korea). Real-time PCR was conducted using the QuantiSpeed SYBR NO-ROX Kit (PhileKorea, Seoul, Republic of Korea) on a Rotor-Gene system (Qiagen, Hilden, Germany). Primers used included GAPDH: Fwd 5′-ACAGCCTCAAGATCA TCAGCAAT-3′, Rev 5′-AGGAAATGAGCTTGACAAAGTGG-3′; IL-1β: Fwd 5′-GGG ATAACGAGGCTTATGTGC-3′, Rev 5′-AGGTGGAGAGCTTTCAGTTCA-3′; TNF-α: Fwd 5′-CAGAGGGCCTGTACCTCATC-3′, Rev 5′-GGAAGACCCCTCCCAAGATAG-3′; MMP-2: Fwd 5′-TTGACGGTAAGGACGGACTC-3′, Rev 5′-ACTTGCAGTACTCC CCATCG-3; and MMP-9: Fwd 5′-TTCCAAACCTTTGAGGGCGA-3′, Rev 5′-CAAAGGCGT CGTCAATCACC-3′.

### 4.9. Intracellular ROS Detection

ROS generation was measured using the DCFDA Cellular ROS Detection Kit (Abcam). For flow cytometry, cells were seeded into 60 mm dishes, stained with 25 µM DCFDA for 45 min at 37 °C, washed, and analyzed using a Cytomics FC500 MLP cytometer (Beckman Coulter, Brea, CA, USA).

For microscopy, DCFDA-stained cells were counterstained with MitoTracker dye for 20 min at 37 °C. Fluorescence images were captured using a fluorescence microscope (Olympus, CKX53, Tokyo, Japan).

### 4.10. Western Blot

Cells were lysed in 1% Triton X-100, and the protein concentration was determined using the Bradford assay (Bio-Rad, Hercules, CA, USA). Equal amounts of protein (20 µg) were resolved by SDS-PAGE and transferred to PVDF membranes. After blocking with 5% BSA in TBST, membranes were incubated overnight at 4 °C with primary antibodies, followed by HRP-conjugated secondary antibodies for 30 min at room temperature. Protein bands were visualized using the ECL solution (GenDEPOT, Katy, TX, USA) and detected with a ChemiDoc MP imaging system (Bio-Rad).

### 4.11. Zymography

After treatment, the medium was collected and subjected to a gelatin zymography assay as described by the manufacturer’s direction (Cosmo Bio, Tokyo, Japan). Briefly, samples were mixed with equivalent amounts of sample buffer and incubated at room temperature for 15 min. Samples were separated on pre-casted acrylamide gels. After electrophoresis, gels were washed with washing buffer at room temperature followed by incubation with a reaction buffer at 37 °C for 36 h. The gels were then incubated with staining solution for 30 min at room temperature, and gelatinase activity was determined as non-stained bands.

### 4.12. Statistical Analysis

All experiments were performed in triplicate or more. Data are expressed as mean ± standard deviation (SD). Statistical significance was determined using one-way ANOVA followed by Tukey’s post hoc test (SPSS v12.0 for Windows). A *p*-value < 0.05 was considered statistically significant.

## 5. Conclusions

This study confirms the potent anti-inflammatory and antioxidant effects of AS632 and AS633 in in vitro models of arthritis. AS632 and AS633 significantly reduced the secretion of key pro-inflammatory cytokines (TNF-α, IL-1β), intracellular ROS accumulation, and MMP expression. Furthermore, it protected chondrocytes from cytokine-induced cytotoxicity. These multifaceted actions suggest that AS632 and AS633 hold potential as a novel topical or systemic therapeutic agent for managing chronic inflammatory disorders such as arthritis.

## Figures and Tables

**Table 1:** Particle sizes and zeta potentials of AS632 and AS633.

Sample	Measurement Type	Replicate 1	Replicate 2	Replicate 3
AS632	Particle size (nm)	238.6	236.4	241.9
	Zeta potential (mV)	−42.7	−48.7	−47.5
AS633	Particle size (nm)	217.0	235.8	227.4
	Zeta potential (mV)	−52.8	−50.2	−47.0

## Data Availability

The original contributions presented in this study are included in the article/Supplementary Materials. Further inquiries can be directed to the corresponding authors.

## Supplementary Materials

The following supporting information can be downloaded at: https://www.mdpi.com/article/10.3390/ph18091270/s1, Table S1: Plant-derived and Synthetic Components in AS632/AS633 Formulations. Refs. [7,8,9,11,12,13,15,17,23,34] cited in Supplementary Materials.

## List of Abbreviations

BSA	Bovine serum albumin
ELISA	Enzyme-linked immunosorbent assay
iNOS	Inducible nitric oxide synthase
LPS	Lipopolysaccharide
MMP	Matrix metalloproteinase
MSM	Methylsulfonylmethane
NSAIDs	Nonsteroidal anti-inflammatory drugs
Peptiskin^®^	Synthetic peptides
ROS	Reactive oxygen species
TNF	Tumor necrosis factor
