# Supplementary material for: Anti-Inflammatory and Antioxidant Effects of Topical Formulations Containing Plant Extracts, Methylsulfonylmethane, and Peptiskin® in In Vitro Models of Arthritis
Source: Pharmaceuticals (Basel). 2025 Aug 26;18(9):1270. doi: 10.3390/ph18091270 (PMC12472448; doi:10.3390/ph18091270)
Supplement: Supplementary file 1 [file pharmaceuticals-18-01270-s001.zip › pharmaceuticals-3694899-supplementary.pdf]

Supplementary Table S1. Plant-derived and Synthetic Components in AS632/AS633 Formulations

Ingredient	Plant Part Used	Extraction Method	Key Actives	Analytical Standard	Reference
Punica granatum seed oil	Seeds	Cold-pressed mechanical extraction	Punicic acid, phytosterols, polyphenols	GC-MS	[1, 2, 3] manufacturer COA
Gaultheria procumbens oil	Leaves	Steam distillation after fermentation (≥3 days)	Methyl salicylate (≥96%)	USP HPLC	[4, 5]
Centella asiatica extract	Whole plant	Ethanol-water maceration and filtration	Asiaticoside, madecassoside, triterpenoids	USP HPLC	[6]
Camellia sinensis leaf extract	Leaves	Water–ethanol extraction and filtration	EGCG, ECG, caffeine	UV-Vis (540 nm)	[7]
MSM	Synthetic (DMSO-derived)	Oxidation of DMSO with H_2O_2, distillation	Methylsulfonylmethane (≥99.8%)	USP GC	[8]
Peptiskin®	Synthetic peptides	Enzymatic synthesis (solvent-free biocatalysis)	Arginine/Lysine oligopeptides	UV at 280 nm	[9, 10]

MSM, Methylsulfonylmethane; COA, Certificate of Analysis; EGCG, Epigallocatechin Gallate; ECG, Epicatechin Gallate; USP, United States Pharmacopeia; GC, Gas Chromatography; HPLC, High Performance Liquid Chromatography; UV-Vis, Ultraviolet–Visible Spectrophotometry; MMPs, Matrix Metalloproteinases.

References:

1. Lansky, E. P., & Newman, R. A. (2007). Punica granatum (pomegranate) and its potential for prevention and treatment of inflammation and cancer. Journal of Ethnopharmacology, 109(2), 177–206.
2. Saeed, M., Naveed, M., Bibi, J., Kamboh, A.A., Arain, M.A., Shah, Q.A., Alagawany, M., El-Hack, M.E.A., Abdel-Latif, M.A., Yatoo, M.I., Tiwari, R., Chakraborty, S., Dhama, K. The promising pharmacological effects and therapeutic/medicinal applications of Punica Granatum L. (Pomegranate) as a functional food in humans and animals, *Recent Patents on Inflammation & Allergy Drug Discovery*. **2018**, 12, 24-38.
3. Rakhshandeh, H., Rahimi, V.B., Habibi, Z., Sirousi, Z., Askari, V.R. Punica granatum seed oil detracts peritoneal adhesion: Perusing antioxidant, anti-inflammatory, antifibrotic, and antiangiogenic impacts, *Physiol. Rep*. **2022**, 10:e15545.

4. Michel, P., Żbikowska, H.M., Rudnicka, K., Gonciarz, W., Krupa, A., Gajewski, A., Machała, P. and Olszewska, M.A. Anti-inflammatory, antioxidant and photoprotective activity of standardised Gaultheria procumbens L. leaf, stem, and fruit extracts in UVA-irradiated human dermal fibroblasts. *Journal of Ethnopharmacology*, **2024,** *319*, p.117219.
5. Michel, P., Wajs-Bonikowska, A., Magiera, A., Wosiak, A., Balcerczak, E., Czerwińska, M.E., Olszewska, M.A. Anti-Inflammatory and antioxidant effects of (6S,9R)-vomifoliol from Gaultheria procumbens L.: In vitro and ex vivo study in human immune cell models, *Int. J. Mol. Sci*. **2025**, 26, 1571.
6. Kumari, S., Deori, M., Elancheran, R., Kotoky, J. and Devi, R. In vitro and in vivo antioxidant, anti-hyperlipidemic properties and chemical characterization of Centella asiatica (L.) extract. *Frontiers in pharmacology*, **2016,** *7*, p.400.
7. Kim, M.J., Yang, Y.J., Min, G.Y., Heo, J.W., Son, J.D., You, Y.Z., Kim, H.H., Kim, G.S., Lee, H.J., Yang, J.H. and Park, K.I. Anti-inflammatory and antioxidant properties of Camellia sinensis L. extract as a potential therapeutic for atopic dermatitis through NF-κB pathway inhibition. *Scientific Reports*, **2025,** *15*(1), p.2371.
8. Colletti, A., Cicero, A.F.G. Nutraceutical approach to chronic osteoarthritis: from molecular research to clinical evidence, *Int. J. Mol. Sci.* **2021**, 22, 12920.
9. Huang, S., Chen, Y., Qiao, Z., Hu, Y., Zhang, H., Zhang, X., Huang, Y. and Xiang, Q. Efficacy of a Novel Lyophilized Arginine Lysine Polypeptide in the Treatment of Acne: In Vitro Experiments and a Split Face Controlled Clinical Trial, J. Biomed. Sci. Res., 2021, 3(1): 132.
10. Solabia Group "Peptiskin® - Active Ingredient - Cosmetics." Solabia Website (Peptiskin® - Active Ingredient - Cosmetics) **2022**.
